# The Effects of Almond Consumption on Cardiovascular Health and Gut Microbiome: A Comprehensive Review

**DOI:** 10.3390/nu16121964

**Published:** 2024-06-20

**Authors:** Saiful Singar, Saurabh Kadyan, Cole Patoine, Gwoncheol Park, Bahram Arjmandi, Ravinder Nagpal

**Affiliations:** 1 The Gut Biome Lab, Department of Health, Nutrition, and Food Sciences, College of Education, Health, and Human Sciences, Florida State University, Tallahassee, FL 32304, USA; ssingar@fsu.edu (S.S.); skadyan@fsu.edu (S.K.); cjp23a@fsu.edu (C.P.); gp21p@fsu.edu (G.P.); 2 Center for Advancing Exercise and Nutrition Research on Aging, Department of Health, Nutrition, and Food Sciences, College of Education, Health, and Human Sciences, Florida State University, Tallahassee, FL 32304, USA

**Keywords:** almond, cardiovascular health, gut microbiota, microbiome, nuts, endothelial function

## Abstract

The consumption of almonds has been associated with several health benefits, particularly concerning cardiovascular and intestinal health. In this comprehensive review, we compile and deliberate studies investigating the effects of almond consumption on cardiovascular disease (CVD) risk factors and gut health. Almonds are rich in monounsaturated fats, fiber, vitamins, minerals, and polyphenols, which contribute to their health-promoting properties. Regular intake of almonds has been shown to improve lipid profiles by reducing LDL cholesterol and enhancing HDL functionality. Additionally, almonds aid in glycemic control, blood pressure reduction, and chronic inflammation amelioration, which are critical for cardiovascular health. The antioxidant properties of almonds, primarily due to their high vitamin E content, help in reducing oxidative stress markers. Furthermore, almonds positively influence body composition by reducing body fat percentage and central adiposity and enhancing satiety, thus aiding in weight management. Herein, we also contemplate the emerging concept of the gut–heart axis, where almond consumption appears to modulate the gut microbiome, promoting the growth of beneficial bacteria and increasing short-chain fatty acid production, particularly butyrate. These effects collectively contribute to the anti-inflammatory and cardioprotective benefits of almonds. By encompassing these diverse aspects, we eventually provide a systematic and updated perspective on the multifaceted benefits of almond consumption for cardiovascular health and gut microbiome, corroborating their broader consideration in dietary guidelines and public health recommendations for CVD risk reduction.

## 1. Introduction

The prevalence of cardiovascular disease (CVD) in the United States is a significant public health concern. According to the US Preventive Services Task Force (USPSTF), CVD represents the leading cause of mortality among adults in the United States, resulting in one out of every three deaths annually [1]. The burden of CVD increases with age, and prevalence varies by race/ethnicity. For instance, in 2015, the adjusted rate of coronary artery disease (CAD) in the United States for adults between 45 and 64 years was 6.1%. This rate increased to 16.4% for individuals aged 65 to 74 years and further rose to 23.3% for those aged 75 and above. The prevalence of CAD was twice as high in American Indian/Alaskan Native adults compared to Asian adults, with rates of 9.3% and 3.7%, respectively [1]. Projections based on the 2020 Census estimates suggest that by 2060, there will be notable rises in the prevalence of CVD and its contributing factors, with minority groups being affected at a higher rate [2]. The American Heart Association (AHA) forecasts that by the year 2030, an estimated 40.5% of the U.S. population will be affected by cardiovascular diseases [3]. These projections underscore the importance of effective prevention strategies and public health interventions to manage the growing burden of CVD, particularly among underserved populations [4].

**Figure 1:**
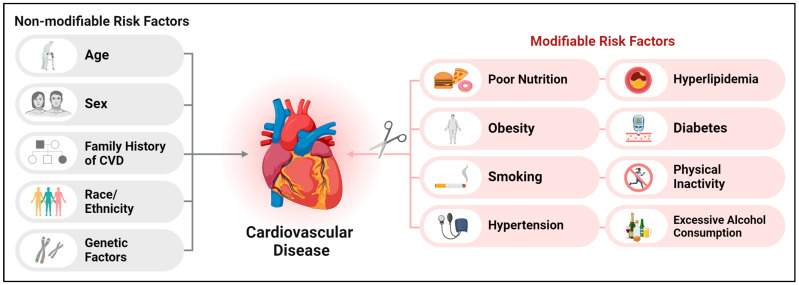
An illustrative depiction of the modifiable and non-modifiable risk factors associated with cardiovascular diseases.

It is widely recognized that the major modifiable risk factors for CVD include hyperlipidemia, hypertension, obesity, diabetes, smoking, and physical inactivity (Figure 1). In the United States, it is estimated that 47% of adults are affected by hypertension, and this number increases to about 70% for individuals aged 65 and over [5]. The prevalence of hyperlipidemia among US adults aged 20 to 44 years decreased from 40.5% in 2009–2010 to 36.1% in 2017–2020 [6]. For diabetes, the age-standardized prevalence rate saw a significant rise, from 9.8% during the 1999–2000 period to 14.3% in the years 2017–2018 [7]. Regarding obesity, data from the National Health and Nutrition Examination Survey (NHANES) for the years 2017–2018 indicate that 42.4% of adults in the United States were affected by obesity [8]. These risk factors are known to contribute significantly to the burden of cardiovascular disease and are the focus of many public health and clinical interventions aimed at reducing cardiovascular morbidity and mortality. Monitoring trends in these risk factors is essential for guiding prevention strategies and healthcare policies.

While several earlier studies have reviewed the effects of almond consumption on CVD risk factors and gut health, this manuscript provides a novel perspective and comprehensive review by integrating several key areas including a detailed discussion of the nutrient composition and polyphenol content of almonds, thereby highlighting their unique properties that contribute to human health benefits. In addition to discussing the impacts of almond consumption on traditional CVD risk factors, this review delves into the effects on endothelial function, oxidative stress, and chronic low-grade inflammation, which are critical markers of cardiovascular health. Furthermore, this manuscript examines the influence of almonds on satiety, appetite measures, and body composition, providing a holistic view of their potential benefits. Moreover, we explore the potential roles of almonds in gut–heart axis modulation, an emerging area of interest in cardiovascular research. By encompassing these diverse aspects, this manuscript aims to offer a thorough and updated perspective on the multifaceted benefits of almond consumption for cardiovascular health and gut microbiome.

## 2. Nutrient Composition and Polyphenol Content of Almonds

Almonds (*Prunus dulcis*) have a nutrient profile that includes a variety of vitamins and minerals, dietary fiber, and healthy fats. They are particularly rich in monounsaturated fats (predominantly oleic acid), which are associated with cardiovascular benefits [9,10,11,12]. Almonds are also a good vitamin E, magnesium, and potassium source [13]. Consuming a serving (approximately 28 g) of almonds delivers 7.3 mg of vitamin E, 77 mg of magnesium, and 208 mg of potassium. These quantities fulfill 48.7%, between 18.3–24.1%, and between 6.1–8.0% of the daily recommended intake for these nutrients in adults, respectively (Table 1). Regarding their polyphenol content, almonds contain a range of bioactive compounds, including flavonoids and phenolic acids. The amount of phenolic compounds found in the skins of almonds can greatly differ between various strains, showing a variation from 127 to 241 milligrams of gallic acid equivalents for every 100 g of fresh weight [14,15,16]. The predominant flavonoids in almonds include isorhamnetin-3-O-galactoside, isorhamnetin-3-O-rutinoside, kaempferol-3-O-rutinoside, epicatechin, quercetin-3-O-galactoside, and catechin [14,17,18,19]. The skins of almonds are recognized for their high concentration of polyphenols, primarily consisting of abundant flavanols and flavanol glycosides [19,20,21]. The antioxidant qualities of almonds, which are linked to their polyphenol composition, have been evaluated through various assays. These evaluations suggest that the skin of almonds may serve as a significant byproduct for the production of antioxidant components for dietary use [20,22,23,24,25,26]. The content of polyphenols and the level of antioxidant activity in almonds can vary based on the strain of almonds and the year of harvest, as shown by differing results in various studies [17].

## 3. Potential Roles of Almonds in Gut–Heart Axis Modulation

The literature indicates that daily consumption of almonds can positively impact cardiovascular and gut health. The consumption of tree nuts, including almonds, may affect the gut–heart axis through several mechanisms. Almonds contain fiber, unsaturated fats, and other bioactive compounds that can influence gut microbiota composition and functionality. Specifically, almond consumption has been linked to higher levels of butyrate production, a type of short-chain fatty acid (SCFA) that is good for the health of the colon. This may lead to a decrease in systemic inflammation and could positively affect heart health [27]. The direct impact of almond consumption on the gut–heart axis is not fully elucidated in the literature, and the precise mechanisms by which almonds may influence this axis remain to be clarified. Hence, the present review aims to comprehensively discuss the benefits of almond consumption on cardiovascular health and gut microbiota, encouraging a broader consideration of almonds in dietary guidelines and public health recommendations for CVD risk reduction.

## 4. Impacts of Almond Consumption on Cardiovascular Health and Associated Risk Factors

### 4.1. Lipid Profile Improvement

Almond consumption has been associated with improvements in lipid profiles, which are important for cardiovascular health. Clinical studies have demonstrated that daily intake of almonds can reduce the levels of low-density lipoprotein cholesterol (LDL-C) and non-high-density lipoprotein cholesterol (non-HDL-C), while maintaining or increasing the levels of high-density lipoprotein cholesterol (HDL-C) [28,29,30,31,32,33,34]. These changes are beneficial, as elevated LDL-C and non-HDL-C are recognized risk factors for CVD, and higher levels of HDL-C are protective. The lipid-modifying effects of almonds are attributed to their nutrient composition, which includes fiber, monounsaturated fatty acids (MUFAs), polyunsaturated fatty acids (PUFAs), and bioactive compounds. Substituting a high-carbohydrate snack with almonds has demonstrated an ability to alter the composition of serum fatty acids. This includes a rise in oleic acid and MUFA levels within serum triglycerides and non-esterified fatty acids. These changes are linked to a decrease in lipid risk factors for CVD and a reduced overall estimated risk of coronary heart disease over a 10-year period [10]. Furthermore, research indicates that almonds can enhance the functionality of HDL, which encompasses the ability to transport cholesterol away from cells. Additionally, almonds positively modify HDL subclasses, boosting the presence of the larger, more beneficial α-1 HDL particles among normal-weight individuals with high LDL-C levels [30]. These functional improvements in HDL may contribute to the cardioprotective effects of almonds. Incorporating almonds into the diet can be a simple dietary strategy to improve lipid profiles and reduce cardiometabolic risk factors. The specific dosages of almonds studied ranged from 43 g per day to approximately 20% of total energy intake, demonstrating a dose–response relationship with lipid improvements [28,29,30,31,32,33,34].

Almond consumption has been shown to alleviate hyperlipidemia through multiple mechanisms, mainly by improving lipid profiles. As discussed above, research indicates that almonds can notably reduce LDL-C, total cholesterol, and non-HDL-C, while increasing HDL-C levels [29,33,35,36]. The nutritional composition of almonds, which are abundant in MUFAs and PUFAs, plays a critical role in improving lipid profiles. The high MUFA content helps to lower LDL-C and total cholesterol levels [10,35,37]. Furthermore, almonds are rich in dietary fiber and phytosterols, which block cholesterol absorption in the intestines, leading to reduced serum cholesterol levels [35]. Almonds are also a good source of antioxidants like vitamin E (α-tocopherol), which can prevent the oxidation of LDL particles, a key contributor to atherosclerosis, thereby enhancing cardiovascular health [35,37]. Additionally, almonds may affect enzymes involved in cholesterol metabolism, such as β-hydroxy-β-methylglutaryl coenzyme A reductase, which plays a crucial role in cholesterol synthesis, resulting in reduced cholesterol production in the liver [38]. Studies have shown a dose–response relationship, indicating that higher almond intake leads to greater improvements in lipid profiles. For instance, daily consumption of 68 g of almonds has been demonstrated to significantly lower LDL-C and total cholesterol [29]. The lipid-lowering effects of almonds can be attributed to their beneficial fatty acid profile, fiber content, antioxidant properties, and their ability to influence cholesterol metabolism. Regular consumption of almonds, particularly in doses ranging from 50 to 100 g per day, has proven effective in managing hyperlipidemia.

### 4.2. Blood Sugar Regulation

Almond consumption is associated with improved glycemic control, which is a key factor in the management of cardiovascular health, particularly for individuals with type 2 diabetes mellitus (T2DM). Almonds have a low glycemic index, and their addition to a meal containing carbohydrates can reduce the overall glycemic impact of the meal. This effect is dose-dependent, with larger quantities of almonds leading to a greater reduction and a slower rise in postprandial blood glucose levels [39,40,41,42]. The consumption of almonds has also been shown to improve glycemic control in patients with T2DM. A study demonstrated that the inclusion of almonds in the diet of patients with T2DM led to reductions in fasting glucose and insulin levels, as well as improvements in the homeostasis model assessment of the insulin resistance index [43]. Almonds can also reduce postprandial glycemia in individuals with well-controlled T2DM. A randomized crossover study revealed that consuming a standard portion of almonds significantly lowered blood sugar levels after meals in individuals diagnosed with T2DM [44]. In addition, chronic ingestion of almonds has been associated with a reduction in hemoglobin A1c, a long-term marker of glycemic control, in individuals diagnosed with T2DM [44]. The specific dosages of almonds that have been studied vary, but beneficial effects on glycemic control have been observed with daily consumption ranging from a standard serving (approximately 28 g) to higher amounts, such as 60 g per day [43,44]. It is important to note that while almonds can be beneficial for blood sugar regulation, they should be incorporated into the diet in a way that maintains overall energy balance to avoid potential weight gain, which could negatively impact glycemic control [45].

Incorporating almonds into the diet can help reduce the risk and manage T2DM through multiple mechanisms. Almond consumption has been shown to lower fasting blood glucose and glycated hemoglobin (HbA1c) levels. A systematic review and meta-analysis revealed that diets including almonds significantly decreased HbA1c levels in patients with T2DM [46]. Another study found that consuming about 60 g of almonds daily improved fasting glucose and insulin resistance indices [43]. Almonds can also help reduce postprandial glucose excursions. A dose–response study indicated that adding almonds to a carbohydrate-rich meal progressively lowered the meal’s glycemic index. This effect is likely due to the high fiber and fat content in almonds, which slow down carbohydrate absorption and mitigate postprandial glucose spikes [39]. Additionally, almond intake is linked to improved insulin sensitivity. Research has shown that meals containing almonds result in lower postprandial glycemia and insulinemia, and increased postprandial insulin sensitivity [47]. Almonds also encourage the growth of short-chain fatty acid (SCFA)-producing gut microbiota, which can enhance glycometabolism. A systematic review indicated that almond-based diets significantly increased beneficial gut bacteria, which are linked to better glycemic control [46]. Moreover, almonds are rich in MUFAs, PUFAs, fiber, and antioxidants, which together contribute to improved glycemic control. These nutrients help reduce oxidative stress and inflammation, both of which play a role in the development of T2DM [43,46]. Regular consumption of almonds, particularly in doses around 60 g per day, can enhance glycemic control, reduce postprandial glucose levels, improve insulin sensitivity, and positively influence gut microbiota, thereby supporting the management of T2DM.

### 4.3. Antioxidant Increase

Almond consumption can increase antioxidants in the body, which is beneficial for cardiovascular health. Almonds are a rich source of vitamin E, an important lipid-soluble antioxidant that protects cells from oxidative damage by scavenging free radicals. Studies have demonstrated that almond consumption can increase the concentrations of plasma α-tocopherol, which is a form of vitamin E [25,26]. For instance, daily consumption of almonds (50 g) for 4 weeks significantly elevated plasma α-tocopherol/cholesterol ratios in healthy middle-aged and young men [25]. Similarly, a randomized clinical trial in Korean adults found that consuming almonds (56 g) daily for 4 weeks increased plasma α-tocopherol by 8.5% [26]. Additionally, almonds contain other antioxidant compounds, such as phenolic acids and flavonoids, which may contribute to their overall antioxidant capacity. Almond consumption has been linked to reduced oxidative stress biomarkers, such as malondialdehyde (MDA) and urinary isoprostanes, which are indicators of lipid peroxidation [27,28]. For example, a study with male smokers supplementing their diet with almonds (84 g) daily for 4 weeks showed significant decreases in lipid peroxidation and oxidative DNA damage, as well as increases in the activities of superoxide dismutase (SOD) and glutathione peroxidase (GPX), which are two important antioxidant enzymes [27]. These findings suggest that almond intake can improve the body’s antioxidant defenses and may help ameliorate oxidative stress, which is a contributing factor to CVD development. The dosages of almonds that have been studied and shown to have beneficial effects on antioxidant status range from 50 to 84 g per day.

### 4.4. Blood Pressure Reduction

Almond consumption has been associated with reductions in blood pressure, which is a significant cardiovascular health benefit. The mechanisms by which almonds may reduce blood pressure are not fully elucidated but may include the presence of heart-healthy monounsaturated fats, magnesium, and potassium, which are known to be involved in blood pressure regulation. The study by Dhillon et al. found that overweight or obese adults who consumed an almond-enriched diet as part of an energy-restricted diet experienced greater reductions in diastolic blood pressure compared to those on a nut-free diet [48]. Additionally, Jenkins et al. reported that compliance with almond intake was significantly related to reductions in both systolic and diastolic blood pressure in hyperlipidemic subjects following a dietary portfolio rich in cholesterol-lowering foods, including almonds [49]. The dosage of almonds associated with blood pressure reduction varied across studies. In the study by Dhillon et al., participants consumed 15% of their energy intake from almonds, while in the study by Jenkins et al., subjects were advised to consume 22.5 g per 1000 kcal of their diet [48,49].

A systematic review and meta-analysis revealed that almond consumption significantly lowered diastolic blood pressure (DBP) but did not have a notable impact on systolic blood pressure (SBP) [50]. Similarly, another study reported that almond consumption led to a weighted mean difference (WMD) of −1.30 mmHg in DBP reduction, without significantly affecting SBP [51]. The subgroup analysis in this study suggested that lower almond doses and participants with lower initial SBP might experience a decrease in SBP. These findings suggest that including almonds in a balanced diet may contribute to blood pressure reduction, which is an important factor in preventing and managing CVD. However, it is important to consider the overall dietary context and energy balance when incorporating almonds into the diet for cardiovascular benefits.

Almond consumption has been linked to lower hypertension through several mechanisms. Firstly, almonds are high in essential nutrients like magnesium, potassium, and arginine, which are vital for vascular health. Magnesium and potassium aid in vasodilation and maintaining electrolyte balance, which can reduce blood pressure [52]. Arginine, as a precursor to NO, enhances endothelial function and decreases vascular resistance due to its vasodilatory properties [53]. Secondly, almonds have been shown to improve lipid profiles by lowering total cholesterol, LDL cholesterol, and apolipoprotein B levels. This improvement can indirectly benefit blood pressure by reducing atherosclerotic burden and enhancing arterial compliance [50,54]. Thirdly, almonds have antioxidant properties that reduce oxidative stress, a contributor to hypertension. By mitigating oxidative stress, NO bioavailability is preserved, further supporting vasodilation and blood pressure regulation [53,55]. Additionally, almonds can help reduce body weight and truncal fat, which are significant risk factors for hypertension. Weight and fat reduction can lead to improved blood pressure by lessening the heart’s workload and enhancing metabolic health [48]. The antihypertensive effects of almonds are multifaceted, involving enhancements in vascular function, lipid profiles, antioxidant status, and body composition. Regular almond consumption, especially in amounts exceeding 42.5 g per day, has been associated with these health benefits.

### 4.5. Chronic Low-Grade Inflammation Amelioration

Almond consumption may decrease chronic low-grade inflammation, a recognized CVD risk factor. The study by Rajaram et al. found that compared to a control diet, a high-almond diet, which replaced 10% or 20% of the energy from a control diet with almonds, resulted in significantly lower serum E-selectin. E-selectin is an adhesion molecule expressed by endothelial cells, and its lower levels suggest a potential anti-inflammatory effect of almonds. Furthermore, levels of C-reactive protein (CRP), an acknowledged indicator of systemic inflammation, were reduced in the groups consuming almond-based diets relative to the control group. However, a distinct dose–response relationship was not evident for either E-selectin or CRP [31]. Additionally, the research conducted by Chen et al. suggested that the intake of almonds appeared to lower vascular cell adhesion molecule-1 (VCAM-1) levels by 5.3% and boost urinary nitric oxide by 17.5%. However, it was noted that these variations did not achieve statistical significance. VCAM-1 is another molecule involved in leukocyte adhesion to the vascular endothelium, and its reduction may indicate an anti-inflammatory effect. The increase in urinary nitric oxide, a vasodilator, could also suggest improved endothelial function [32]. The dosage of almonds in these studies varied, with Rajaram et al. using a 10% or 20% isocaloric replacement of a control diet with almonds and Chen et al. incorporating 85 g of almonds daily into the diet [31,32]. These findings suggest that almond consumption may benefit markers of inflammation, which is important for cardiovascular health. However, further research is needed to establish a clear dose–response relationship and understand these anti-inflammatory effects’ underlying mechanisms.

### 4.6. Endothelial Function Improvement

Almond consumption has been associated with improvements in endothelial function, which is a key factor in cardiovascular health. Endothelium, the inner lining of blood vessels, plays an important role in vascular homeostasis, including the regulation of vascular tone and blood flow. Endothelial dysfunction is a precursor to atherosclerosis and is predictive of cardiovascular events. A randomized controlled trial (ATTIS Study), showed that adults at a heightened risk for CVD experienced enhanced endothelium-dependent vasodilation, as indicated by improvements in flow-mediated dilation (FMD), following the intake of whole roasted almonds [56]. This improvement in endothelial function was significant, with a mean difference of 4.1% units in FMD compared to control snacks. Additionally, almond consumption was shown to lower LDL-C, although no effects were observed on liver fat or other cardiometabolic risk factors [56]. Another study found that almond consumption did not significantly improve vascular function, as measured by FMD, peripheral arterial tonometry (PAT), and pulse wave velocity (PWV) in patients with CAD [57]. However, the study did observe a tendency for almonds to decrease VCAM-1 and increase urinary nitric oxide levels, which are indicative of improved endothelial function. Almonds are rich in fiber, MUFAs, α-tocopherol, and other bioactive compounds that may contribute to their beneficial effects on endothelial function. The presence of α-tocopherol, for instance, has been shown to improve vascular function and reduce oxidative stress [58]. Additionally, almonds have been reported to inhibit dyslipidemia and vascular dysfunction by inhibiting cholesterol synthesis, protecting endothelial nitric oxide synthase, and promoting nitric oxide release [38]. Almond consumption may improve endothelial function through a combination of improved vasodilation, reduced oxidative stress, and favorable changes in lipid profiles. However, the extent of these benefits can vary depending on individual health status and the presence of other CVD risk factors.

### 4.7. Body Composition Improvement

Almond consumption has been associated with improvements in body composition, particularly in the context of cardiovascular health. The literature suggests that almonds can beneficially affect adiposity, which is a component of body composition relevant to cardiovascular risk. In a 12-week randomized crossover clinical trial, daily supplementation of ~60 g of almonds to the diet of patients with T2DM and mild hyperlipidemia was found to significantly lower body fat percentage as determined by bioelectrical impedance analysis [43]. This study also reported improvements in glycemic control and lipid profiles, which are important factors in cardiovascular health. Another randomized controlled trial found that daily consumption of 1.5 oz of almonds, replacing a high-carbohydrate snack, reduced abdominal and leg fat in healthy adults with dyslipidemia despite no differences in total body weight [28]. This suggests that almonds may help redistribute body fat or reduce fat in specific areas, which is relevant since central adiposity is a known risk factor for cardiometabolic diseases. Furthermore, a study examining the effects of almond consumption on body weight in habitual snackers over a 12-month period found that compared with a popular discretionary snack food, incorporating almonds into the diet improved diet quality without significant changes in body weight [59]. These findings indicate that almond consumption may improve body composition by reducing body fat percentage and central adiposity, which are important considerations for cardiovascular health. The dosages of almonds used in these studies ranged from 1.5 oz to 60 g per day. It is important to consider these findings in the context of an overall healthy diet and lifestyle for the management of cardiovascular risk.

Almond consumption can help mitigate obesity through multiple mechanisms. Almonds are rich in nutrients, high in fiber, protein, and healthy fats, which enhance satiety and decrease overall caloric intake. Research indicates that adding almonds to one’s diet can result in reduced hunger and lower energy intake afterward [60,61]. The high fiber content of almonds can decrease the bioavailability of macronutrients, thereby reducing net metabolizable energy. This reduction in energy absorption efficiency aids in weight control [62]. Clinical trials have shown that almond consumption, especially within an energy-restricted diet, significantly reduces body fat, including truncal and visceral adipose tissue. For example, a study demonstrated that incorporating almonds into a diet with a 500-kcal deficit led to more significant reductions in truncal and total body fat compared to a diet without nuts [48]. Almonds also positively influence gut microbiota composition by increasing beneficial bacterial taxa. This alteration in gut microbiota can enhance metabolic health and support weight management [63,64]. Moreover, almond consumers often have higher diet quality scores, characterized by increased intake of essential nutrients and reduced intake of harmful components like trans-fatty acids and sugars. This overall dietary improvement can aid in managing weight and reducing the risk of obesity [65]. Regular almond consumption can assist in reducing obesity through mechanisms such as improved satiety, decreased energy absorption, body fat reduction, enhanced gut microbiota, and better overall diet quality.

### 4.8. Appetite and Satiety Regulation

Almond consumption may regulate appetite and satiety through its effects on appetite-regulating hormones and the physical properties of the nuts themselves. Research conducted by Carter et al. demonstrated that almond consumption resulted in more positive responses from hormones that regulate appetite after meals. This included a reduced C-peptide area under the curve (AUC) and increased AUC responses for the glucose-dependent insulinotropic polypeptide, glucagon, and pancreatic polypeptide, in contrast to the effects of a snack bar high in carbohydrates [60]. However, these hormonal changes were not associated with self-reported appetite or short-term energy consumption. Tan and Mattes reported that almonds eaten as snacks reduced hunger and desire to eat during an acute feeding session [42]. This effect was most prominent when almonds were eaten as snacks rather than with meals. The study by Hull et al. also supported the notion that almonds can enhance satiety, as meal consumption during lunch and dinner was significantly reduced in a dose-dependent manner in response to almond snacks, without a net increase in energy consumed over the day [66]. Almonds are high in dietary fiber, protein, and healthy fats, which are macronutrients known to promote satiety. The physical properties of almonds, such as their crunchiness and the need to chew, may also contribute to satiety by increasing oral sensory signals that can influence satiation [67,68,69,70,71,72,73]. The dosages of almonds used in these studies varied, with amounts ranging from 28 g to 50 g or 1.5 oz, typically representing a portion size that could be reasonably included in a daily diet for satiety regulation. It is important to consider these findings in the context of an overall balanced diet and to account for the energy content of almonds when incorporating them into dietary patterns for weight management and cardiovascular health.

### 4.9. Gut Microbiome Modulation

Almond consumption has been shown to have several beneficial effects on the gut microbiome. A randomized controlled trial conducted by Holscher et al. revealed that almond intake increased the relative abundances of several beneficial bacterial genera including *Lachnospira*, *Roseburia*, and *Dialister* [74]. The study highlighted that the form of almond processing (e.g., whole, roasted, chopped, or almond butter) influenced these bacterial populations differently [74]. Specifically, chopped almonds significantly boosted the relative abundances of *Lachnospira*, *Roseburia*, and *Oscillospira*, whereas whole almonds particularly enhanced *Dialister* levels. In contrast, almond butter did not produce significant changes in these bacterial populations compared to the control group, suggesting that the physical form of almonds plays a crucial role in their impact on gut microbiota. The absence of significant changes with almond butter could be due to differences in the bioavailability of almond components affecting microbial fermentation. The bacterial genera *Lachnospira*, *Roseburia*, *Dialister*, and *Oscillospira* are integral to the regulation of cardiovascular and metabolic health through various mechanisms. *Lachnospira* is known for producing SCFAs, particularly butyrate, which has anti-inflammatory properties and enhances gut barrier function [75]. Increased SCFA production is linked to improved insulin sensitivity and reduced systemic inflammation, both beneficial for cardiometabolic health. *Roseburia* species are major butyrate producers, with anti-inflammatory effects, improved gut barrier integrity, and lipid metabolism modulation [76]. Higher levels of *Roseburia* have been inversely correlated with atherosclerosis and systemic inflammation, indicating a protective role against cardiovascular diseases and metabolic disorders [77]. *Dialister* species, though less studied, are part of the gut microbiota associated with health, contributing to the fermentation of dietary fibers into SCFAs, which enhance metabolic health by improving insulin sensitivity and reducing inflammation [78]. Changes in *Dialister* populations have been associated with metabolic dysregulation, highlighting their potential role in cardiometabolic health [79]. *Oscillospira* is linked to leanness and health, involved in the metabolism of complex carbohydrates, and associated with lower BMI and reduced obesity risk. This genus also plays a role in cholesterol metabolism, positively influencing lipid profiles and reducing cardiovascular risk [80]. These bacterial genera contribute to cardiovascular and metabolic health through SCFA production, anti-inflammatory effects, enhanced gut barrier function, and modulation of lipid and glucose metabolism, essential for maintaining metabolic homeostasis and reducing cardiometabolic disease risk.

Another study by Creedon et al. found that almond consumption, whether whole or ground, resulted in increased levels of butyrate, a microbial-derived SCFA associated with gut health, compared to a control group [27]. This rise in butyrate is attributed to the fermentation of almond fibers by butyrate-producing bacteria within the *Ruminococcaceae* family, which includes genera like *Ruminiclostridium* and *Ruminococcaceae NK4A214*. These bacteria metabolize complex carbohydrates into butyrate [81,82]. Additionally, Mandalari et al. discovered that finely ground almonds significantly boosted populations of *Eubacterium rectale*, a known butyrate-producer, during in vitro fermentation [83]. This indicates that finely ground almonds enhance the availability of fermentable substrates, thereby promoting butyrate production. However, no significant changes were observed in the overall gut microbiome composition or diversity at the phylum level.

Research by Choo et al. showed that regular almond consumption increased the abundance of *Ruminococcaceae*, a beneficial bacterial family, in adults with overweight and obesity and elevated fasting blood glucose [63]. This study also noted significant changes in microbiome composition and increased bacterial richness, evenness, and diversity. Specifically, there were significant increases in the relative and absolute abundances of operational taxonomic units within the *Ruminococcaceae* family, including *Ruminiclostridium*, *Ruminococcaceae NK4A214*, and *Ruminococcaceae UCG-003*. These changes were not observed in the control group, which consumed an isocaloric, high-carbohydrate biscuit snack. The dietary fibers in almonds are fermented by these beneficial bacteria, promoting their growth and activity [84]. This fermentation process produces metabolites that support the proliferation of beneficial bacterial populations [85]. Although the study did not detect changes in fecal SCFAs levels or the genes encoding butyryl-CoA:acetate CoA-transferase, the increase in beneficial *Ruminococci* suggests a favorable shift in the gut microbiome composition that could enhance metabolic health.

Liu et al. reported that consuming almonds and almond skins significantly increased the growth of beneficial *Bifidobacterium* spp. and *Lactobacillus* spp. while reducing the growth of potentially pathogenic *Clostridium perfringens* in healthy adults [86]. These beneficial bacteria utilize the fibers and polyphenols in almonds and almond skins for fermentation, leading to their proliferation [87]. The study also noted that *C. perfringens* was significantly suppressed, likely due to competitive exclusion, where the increased abundance of beneficial bacteria outcompetes pathogenic bacteria for nutrients and adhesion sites in the gut [88,89]. Furthermore, the fermentation of almond fibers by beneficial bacteria results in the production of SCFAs like butyrate, which have antimicrobial properties that can inhibit the growth of pathogenic bacteria [90]. The study also observed changes in bacterial enzyme activities, including increased fecal β-galactosidase activity and decreased activities of β-glucuronidase, nitroreductase, and azoreductase, which are associated with harmful bacterial metabolism. The prebiotic fibers and polyphenols in almonds and almond skins promote the growth of beneficial bacteria like *Bifidobacterium* spp. and *Lactobacillus* spp., while inhibiting pathogenic bacteria such as *C. perfringens* through competitive exclusion and the production of antimicrobial SCFAs.

Almond consumption has also been found to impact the gut microbiome across various preclinical models. One study using a rat model showed that almond supplementation significantly altered the gut microbiome, notably increasing the relative abundance of major gut bacterial phyla Bacteroidetes and Firmicutes in the caecum of rats fed almond beverages [91]. Another study on rats found that both raw and roasted almonds encouraged the growth of beneficial gut bacteria like *Bifidobacterium* spp. and *Lactobacillus* spp., while reducing the presence of *Enterococcus* spp. [92]. In-vitro studies also demonstrate almonds’ ability to modulate the gut microbiome. For example, finely ground almonds significantly boosted the populations of *Bifidobacterium* spp. and *Eubacterium rectale*, which was associated with increased butyrate production, indicating a potential prebiotic effect [83]. Another in-vitro study found that almond polysaccharides enhanced the concentration of SCFAs and beneficial bacteria such as *Lactobacillaceae* and *Bifidobacteriaceae* during fecal fermentation [93]. Ex-vivo studies, such as those using porcine fecal fermentation, have indicated that almond substrates can stimulate the growth of specific microbial genera, including *Tyzzerella*, *Lachnospiraceae*, and *Enterococcus*, and increase SCFA concentrations, which are advantageous for gut health [94]. Almond consumption appears to positively affect the gut microbiome by fostering the growth of beneficial bacteria and boosting SCFA production, which are crucial for maintaining gut health.

## 5. Conclusions

**Figure 2:**
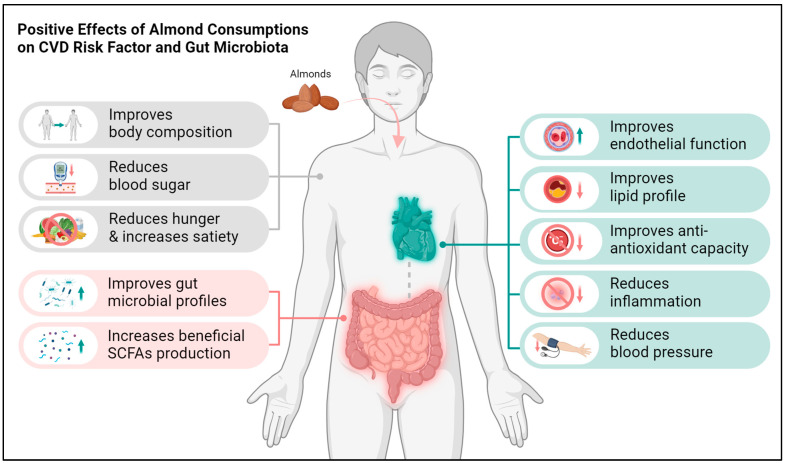
Positive effects of almonds consumption on cardiovascular disease risk factors and gut microbiome. Dotted line represents the gut–heart axis.

The current literature implies that almond consumption has a positive impact on cardiovascular health and can modulate the gut microbiome (Figure 2). Specifically, almond consumption has been associated with improvements in endothelial function, lipid profiles, and body composition, which are important factors in cardiovascular health [27,28,56,57,63]. They have also been associated with reductions in body fat percentage and central adiposity, which are risk factors for cardiometabolic diseases [28,43]. Regarding the gut microbiome, almond consumption has been shown to increase the production of beneficial SCFAs, particularly butyrate, which has anti-inflammatory properties and may play a role in cholesterol metabolism [27]. Almond consumption has also been associated with changes in fecal microbiota composition, including increases in bacterial richness, evenness, and diversity, particularly within the *Ruminococcaceae* family [63]. However, these changes in the microbiota did not translate into changes in fecal SCFA levels in one of the studies [63]. The dosages of almonds used in these studies varied but were typically around 56 g/day. The findings suggest that incorporating almonds into the diet can improve cardiovascular health markers and positively modulate the gut microbiome, potentially contributing to overall health benefits. However, the capacity for these microbiological effects to precipitate host benefit, particularly in relation to cardiovascular health, requires further investigation.

Studies investigating the impact of almond consumption on CVD risk factors have several limitations. One limitation is the variability in study design, including differences in the duration of the studies, the amount of almonds consumed, and the dietary context in which almonds are consumed. For example, some studies may not provide detailed dietary instructions for incorporating almonds into the diet, which can lead to increased energy intake and potential weight gain, as seen in a study where long-term almond consumption was associated with increased BMI and waist circumference [45]. Another limitation is the heterogeneity of study populations, which can include individuals with different health statuses, such as those with T2DM, CAD, or varying degrees of metabolic health. This can affect the generalizability of the results [34,45,57,95,96]. Additionally, the sample sizes in some studies may be small, and the study periods may be too short to detect long-term effects on CVD risk factors [31,57,62]. There is also the issue of compliance and the accuracy of self-reported dietary intake, which can influence the study outcomes [45,62]. Furthermore, the control foods used for comparison in some studies may not be equivalent in macronutrient composition, which can confound the results [31,95]. The potential for publication bias and the lack of blinding in some studies can also affect the validity of the findings [34]. Lastly, while some studies show beneficial effects of almonds on certain lipid profiles, such as LDL-C, they may not show significant effects on other cardiometabolic outcomes, such as blood pressure, HDL-C, and inflammatory markers, indicating a need for further research to fully understand the impact of almonds on CVD risk [31,34,57].

The American College of Cardiology/American Heart Association (ACC/AHA) guidelines on the primary prevention of CVD recommend plant-based and Mediterranean diets, which include nuts, due to their association with lower risk of all-cause mortality and certain CVD outcomes [97]. However, the guidelines also emphasize the need for further research to establish the clinical efficacy of specific dietary components, such as almonds, in the prevention of CVD.

In summary, the current evidence suggests that almonds may improve certain CVD risk factors, particularly LDL-C, and alter gut microbiome composition; although, the clinical implications of these findings, especially regarding the role of specific gut microbes and metabolites in mediating these effects, require further investigation. Specifically, to our knowledge, no evidence was found in the literature regarding the effects of almond consumption on gut microbiota-derived metabolites and their relationship with cardiometabolic risk factors, which should be an important topic for future studies.

## Figures and Tables

**Table 1:** Energy and nutrient comparison chart for almond and other tree nuts per 100 g portion.

Per 100 g Portion	Almond	Brazil Nut	Cashew	Hazelnut	Macadamia	Pecan	Pistachio	Walnut
Energy, kcal	579	659	553	628	718	691	560	654
Protein, g *	21.2	14.3	18.2	15	7.91	9.17	20.2	15.2
Carbohydrate, g	21.6	11.7	30.2	16.7	13.8	13.9	27.2	13.7
Total Dietary Fiber, g *	12.5	7.5	3.3	9.7	8.6	9.6	10.6	6.7
Total Fat, g	49.9	67.1	43.8	60.8	75.8	72	45.3	65.2
Saturated FAs, g *	3.8	16.1	7.78	4.46	12.1	6.18	5.91	6.13
Monounsaturated FAs, g	31.6	23.9	23.8	45.7	58.9	40.8	23.3	8.93
Polyunsaturated FAs, g	12.3	24.9	7.84	7.92	1.5	21.6	14.4	47.2
Minerals								
Calcium, mg *	269	160	37	114	85	70	105	98
Iron, mg	3.71	2.43	6.68	4.7	3.69	2.53	3.92	2.91
Magnesium, mg	270	376	292	163	130	121	121	158
Phosphorus, mg	481	725	593	290	188	277	490	346
Potassium, mg	733	659	660	680	368	410	1020	441
Sodium, mg	1	3	12	0	5	0	1	2
Zinc, mg	3.12	4.06	5.78	2.45	1.3	4.53	2.2	3.09
Copper, mg	1.03	1.74	2.2	1.72	0.756	1.2	1.3	1.59
Manganese, mg	2.18	1.22	1.66	6.18	4.13	4.5	1.2	3.41
Selenium, µg	4.1	1920	19.9	2.4	3.6	3.8	7	4.9
Vitamins								
Vitamin A (RAE), µg	0	0	0	1	0	3	26	1
Thiamin, mg *	0.205	0.617	0.423	0.643	1.2	0.66	0.87	0.341
Riboflavin, mg *	1.14	0.035	0.058	0.113	0.162	0.13	0.16	0.15
Niacin, mg *	3.62	0.295	1.06	1.8	2.47	1.17	1.3	1.12
Pantothenic Acid, mg	0.471	0.184	0.864	0.918	0.758	0.863	0.52	0.57
Pyridoxine, mg	0.137	0.101	0.417	0.563	0.275	0.21	1.7	0.537
Biotin, µg	N/A	N/A	N/A	N/A	N/A	N/A	N/A	N/A
Folate (DFE), µg	44	22	25	113	11	22	51	98
Cobalamin, µg	0	0	0	0	0	0	0	0
Vitamin C, mg *	0	0.7	0.5	6.3	1.2	1.1	5.6	1.3
Vitamin D (D2 + D3), µg	0	0	0	0	0	0	0	0
Vitamin E, mg *	25.6	5.62	0.9	15	0.54	1.4	2.86	0.7
Phylloquinone, µg	0	0	34.1	14.2	N/A	3.5	N/A	2.7

* Almonds contain the highest/lowest amount of this nutrient per 100 g portion compared to other tree nuts. All tree nuts compared, except for Brazil nut, are raw and unsalted. Brazil nuts are dried and unblanched. Data is from the USDA FoodData Central. N/A: Not applicable.
